# A case of late-presenting congenital diaphragmatic hernia diagnosed at 5 years with acute abdomen

**DOI:** 10.1186/s40792-024-01980-0

**Published:** 2024-07-30

**Authors:** Ryuta Masuya, Kazuhiko Nakame, Shun Munakata, Shinsuke Takeno, Atsushi Nanashima, Satoshi Ieiri

**Affiliations:** 1 Division of the Gastrointestinal, Endocrine and Pediatric Surgery, Department of Surgery, Faculty of Medicine, University of Miyazaki, 5200, Kiyotakecho-Kihara, Miyazaki, Miyazaki 889-1692 Japan; 2 Division of Hepato-Biliary-Pancreatic Surgery, Department of Surgery, Faculty of Medicine, University of Miyazaki, 5200, Kiyotakecho-Kihara, Miyazaki, Miyazaki 889-1692 Japan; 3 Department of Pediatric Surgery, Research Field in Medical and Health Sciences, Medical and Dental Area, Research and Education Assembly, Kagoshima University, 8-35-1, Sakuragaoka, Kagoshima, 890-8520 Japan

**Keywords:** Congenital diaphragmatic hernia, Late presentation, Acute abdomen, Pulmonary hypoplasia

## Abstract

**Background:**

Some congenital diaphragmatic hernias are diagnosed beyond 1 month. A late-presenting congenital diaphragmatic hernia shows a variety of clinical manifestations, and the preoperative clinical course is variable. We herein report a pediatric case of late-presenting congenital diaphragmatic hernia diagnosed as acute abdomen.

**Case presentation:**

A 5-year-old boy was brought to our hospital because of herniation of the intestine into the left thoracic cavity, which was observed on radiography performed for abdominal pain. Enhanced computed tomography showed herniation of the small intestine and colon into the left thoracic cavity. Emergency laparoscopic surgery was performed based on the diagnosis of left diaphragmatic hernia. The entire small intestine and part of the colon herniated from the posterolateral defect of the diaphragm. We were able to retract the herniated intestine back into the abdomen but confirmed that the diaphragmatic defect and closure of the defect seemed to be technically challenging via laparoscopy; therefore, we converted the procedure to open laparotomy. The diaphragmatic defect was directly closed with interrupted sutures, and the thoracic cavity was degassed. Postoperatively, the left lung was found to be poorly expanded, but pulmonary hypoplasia was not evident in this case.

**Conclusions:**

We herein report a pediatric case of late-presenting congenital diaphragmatic hernia diagnosed as abdominal pain. Late-presenting congenital diaphragmatic hernias present with a wide variety of symptoms; therefore, it is important to be reminded of these conditions and check chest radiographs in children presenting with acute or chronic respiratory or gastrointestinal symptoms of unknown etiology.

## Backgrounds

Some congenital diaphragmatic hernias (CDH) are diagnosed beyond 1 month. Late-presenting CDH (LPCDH) shows a variety of clinical manifestations [1], and the clinical course leading up to diagnosis varies depending on the patient.

We herein report a case of LPCDH diagnosed as an abdominal pain.

## Case presentation

The patient was a 5-year-old boy. No perinatally specific abnormalities were noted. He had a history of suspected bronchial asthma; however, chest radiography was never performed. At 5 years, he complained to his primary-care pediatrician of abdominal pain. He was transferred to our hospital with a diagnosis of diaphragmatic hernia, because chest and abdominal X-ray revealed a herniated intestine in the left thoracic cavity.

**Fig. 1:**
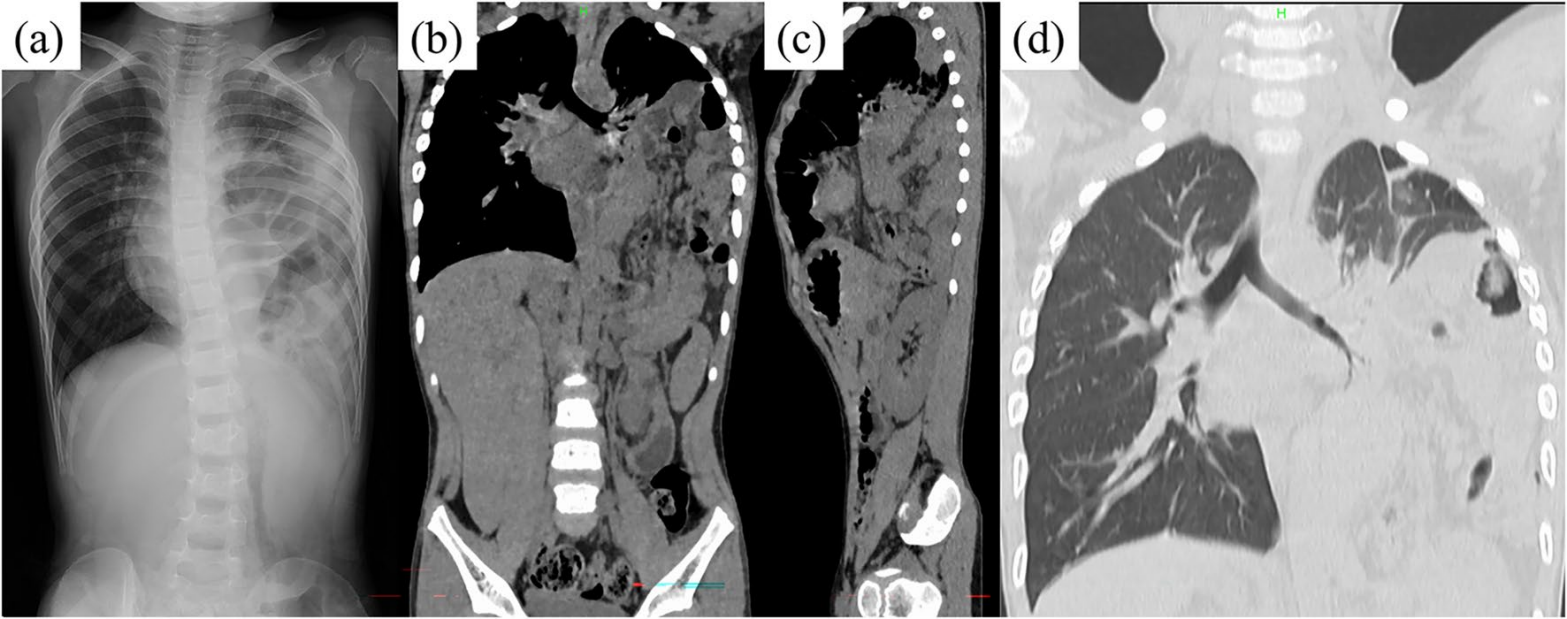
Findings of preoperative imaging. **a** Thoracoabdominal X-ray showed a herniated intestine in the left thoracic cavity and no intestinal gas in the abdomen. The coronal section (**b**) and sagittal section (**c**) and coronal section of pulmonary window setting (**d**) of plain thoracoabdominal CT showed herniation of the small intestine and colon into the left thoracic cavity. The spleen and stomach were not herniated. Atelectasis of left lung was mild

When he visited our hospital, his consciousness was clear, his temperature was 37.0 °C, pulse rate was 144 beats/min, blood pressure was 116/88 mmHg, respiratory rate was 27/min, SpO_2_ was 97% (room air). The abdomen was flat, soft, and mildly tender, but the site of tenderness was unclear. Respiratory sounds were weak on the left side. A blood biochemistry examination revealed no abnormal findings, except for a mildly elevated white blood cell count of 17,700/mm^3^ and C-reactive protein of 2.48 mg/dL. Thoracoabdominal radiography revealed herniated intestine in the left thoracic cavity, and no intestinal gas was detected in the abdomen (Fig. 1a). Plain thoracoabdominal computed tomography (CT) showed herniation of the small intestine and colon into the left thoracic cavity (Fig. 1b–d). There was mild atelectasis.

**Fig. 2:**
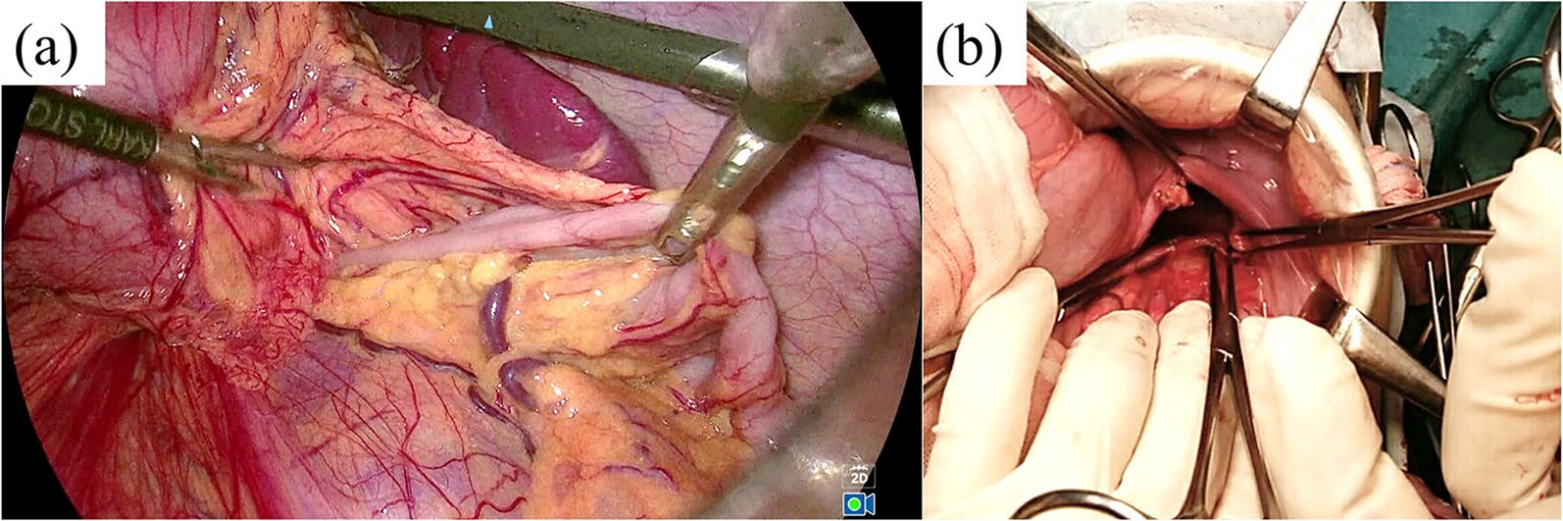
Intraoperative findings. **a** Laparoscopic exploration showed that the small intestine and colon had herniated into the thoracic cavity through the dorsal aspect of the stomach. **b** We opened the abdomen and found a 4.5 × 3 cm defect on the posterolateral part of the left diaphragm

We performed emergency surgery with a diagnosis of a left diaphragmatic hernia. Laparoscopic exploration showed that the entire small intestine and most of the colon had herniated into the thoracic cavity through the posterolateral defect of the diaphragm (Fig. 2a). We were able to retract the intestine back into the abdomen, but the laparoscopic procedure, including the confirmation of the precise size and shape of the diaphragm and following closure seemed to be challenging and difficult, so we converted the procedure to open laparotomy. We found a 4.5 × 3 cm defect on the posterolateral part of the left diaphragm (Fig. 2b) and then closed the defect by a direct interrupted suture using non-absorbable multi-filament thread and then removed the remaining free air from within the left thoracic cavity.

Despite degassing from the left thoracic cavity during the operative procedure, the postoperative expansion of the left lung was insufficient; CT showed no evidence of pulmonary problems, including torsion. The atelectasis was mild. Pulmonary hypoplasia, which may occur as a result of congenital herniation of the intestinal tract, was not evident in this case. The patient’s respiratory status remained good, and the left lung gradually expanded. Echocardiographic findings revealed no pulmonary hypertension. His respiratory and cardiac functions were unaffected for 2 years during the postoperative follow-up period.

## Discussion

LPCDH diagnosed after 1 month shows the following three types of onset: (1) a congenital diaphragmatic defect that was not originally associated with organ herniation, with symptoms appearing when abdominal organs suddenly herniate due to increased abdominal pressure or other causes; (2) congenital herniation into the thoracic cavity, diagnosed when symptoms become apparent; and (3) asymptomatic and discovered incidentally on an examination.

In the present case, respiratory symptoms suggestive of bronchial asthma were noted before the onset of the disease, and the lungs were poorly expanded after repair of the diaphragm, suggesting that the intestinal tract had potentially congenitally herniated into the left thoracic cavity, and abdominal pain led to a definitive diagnosis.

The proportion of LPCDH to total CDH varies among reports [1–4], and this variation may depend on the accuracy of a prenatal diagnosis in the region and the investigation of the causes of neonatal deaths. The proportion of LPCDH to total CDH in the Japanese data reported in 2005 was only 2.6% [2], whereas a report from rural Egypt 2002 showed a very high incidence rate of 45.5% [4].

Bagłaj collected and reviewed 362 cases of LPCDH in 2004 [1]; 79.4% were left-sided, while 20.6% were right-sided, and 72% of left-sided cases were encountered before 1 year, compared with 87% at ≥ 1 year. The percentage of left-sided onset increased with age.

LPCDH presents with a wide variety of clinical manifestations (Table 1). In a previous review [1], 60% of patients on the left side had acute symptoms, 34% had chronic symptoms, and 6% were asymptomatic. In comparison, 32% had acute symptoms, 57% had chronic symptoms, and 11% were asymptomatic among right-sided LPCDH cases. Respiratory symptoms, such as tachypnea, cough, and recurrent airway infections, tend to be more common on the right side than on the left [2]. In contrast, gastrointestinal symptoms, such as abdominal pain and vomiting, were as common as respiratory symptoms on the left side. Gastrointestinal symptoms were less frequently recognized on the right side than on the left side, probably because the herniated liver prevented the prolapse of the intestinal tract. It is also important to note that in the left-sided cases, the frequency of an acute onset was as high as 82% in patients under 1 year [1]. In the present case, abdominal pain was the trigger for the diagnosis, but it is possible that asthma-like respiratory symptoms might be associated with CDH.

**Table 1:** The clinical symptoms in patients with LPCDH

	Left (*n* = 205)	Right (*n* = 42)
Type of presentation	Acute/chronic	Acute/chronic
Symptom		
Dyspnea, tachypnea	72/26	8/10
Cough	24/15	5/8
Vomiting	69/17	9/4
Recurrent respiratory infection	–/19	–/6
Abdominal pain	34/11	4/2
Chest pain	8/2	1/–
Failure to thrive/poor weight gain	–/18	–/9
Irritability	10/2	1/–
Asymptomatic	7	5

The most common clinical symptoms in patients with late-presenting CDH, quoted from the review article by Bagłaj [1]

Data were adequately presented in 205 children with left CDH and 42 with right CDH

Hernia with sacs have been reported to be present in about 10% of cases of LPCDH [1, 2], with no apparent difference from CDH diagnosed in the neonatal period; the presence of a sac may thus have no influence on the presentation of CDH.

In the present case, poor expansion of the left lung after surgery showed gradual improvement over time. Postoperative echocardiography revealed no evidence of pulmonary hypertension. However, congenital pulmonary hypoplasia associated with CDH is expected to result in a relatively underdeveloped vascular bed. Pulmonary blood flow scintigraphy has been reported to help evaluate postoperative pulmonary blood flow in CDH operated in the neonatal period and is an indicator of functional prognosis [5, 6]. However, no reports have investigated the usefulness of pulmonary blood flow scintigraphy for LPCDH, and we did not perform it in the present case.

In recent years, laparoscopic or thoracoscopic surgery has become the surgical option of choice in an increasing number of LPCDH cases [7, 8]. Obata et al. reported little difference in outcomes between laparoscopic and thoracoscopic surgery but pointed out that the thoracoscopic approach may be superior in terms of technical difficulty and frequency of postoperative recurrence [9]. Since the patient presented with abdominal symptoms, we considered the need to search for abnormal findings of the abdomen other than the diaphragmatic hernia and attempted a laparoscopic procedure; however, the intestinal tract retracted back into the abdominal cavity, obstructing the view of the diaphragmatic defect.

## Conclusions

We encountered a case of LPCDH diagnosed as abdominal pain. LPCDH presents with a wide variety of symptoms; therefore, it is important to remember this condition and perform chest radiography in children presenting with acute or chronic respiratory or gastrointestinal symptoms of unknown etiology.

## Data Availability

Not applicable.

## Abbreviations

CDH: Congenital diaphragmatic hernia
LPCBD: Late-presenting congenital diaphragmatic hernia
